# High-quality Population-specific Haplotype-resolved Reference Panel in the Genomic and Pangenomic Eras

**DOI:** 10.1093/gpbjnl/qzaf022

**Published:** 2025-03-10

**Authors:** Qingxin Yang, Yuntao Sun, Shuhan Duan, Shengjie Nie, Chao Liu, Hong Deng, Mengge Wang, Guanglin He

**Affiliations:** Department of Oto-Rhino-Laryngology & Institute of Rare Diseases, West China Hospital of Sichuan University, Sichuan University, Chengdu 610000, China; Center for Archaeological Science, Sichuan University, Chengdu 610000, China; School of Forensic Medicine, Kunming Medical University, Kunming 650500, China; Department of Oto-Rhino-Laryngology & Institute of Rare Diseases, West China Hospital of Sichuan University, Sichuan University, Chengdu 610000, China; Center for Archaeological Science, Sichuan University, Chengdu 610000, China; West China School of Basic Science & Forensic Medicine, Sichuan University, Chengdu 610000, China; Department of Oto-Rhino-Laryngology & Institute of Rare Diseases, West China Hospital of Sichuan University, Sichuan University, Chengdu 610000, China; Center for Archaeological Science, Sichuan University, Chengdu 610000, China; School of Basic Medical Sciences, North Sichuan Medical College, Nanchong 637100, China; School of Forensic Medicine, Kunming Medical University, Kunming 650500, China; Anti-Drug Technology Center of Guangdong Province, Guangzhou 510230, China; School of Forensic Medicine, Kunming Medical University, Kunming 650500, China; Department of Oto-Rhino-Laryngology & Institute of Rare Diseases, West China Hospital of Sichuan University, Sichuan University, Chengdu 610000, China; Faculty of Forensic Medicine, Zhongshan School of Medicine, Sun Yat-sen University, Guangzhou 510275, China; Department of Forensic Medicine, College of Basic Medicine, Chongqing Medical University, Chongqing 400331, China; Human Genetics and Forensic Genomics Research Institute, College of Basic Medicine, Chongqing Medical University, Chongqing 400331, China; Department of Oto-Rhino-Laryngology & Institute of Rare Diseases, West China Hospital of Sichuan University, Sichuan University, Chengdu 610000, China; Center for Archaeological Science, Sichuan University, Chengdu 610000, China

**Keywords:** Haplotype reference panel, Imputation accuracy, Genotype imputation, Population genomics, Genomic medicine

## Abstract

Large-scale international and regional human genomic and pangenomic resources derived from population-scale biobanks and ancient DNA sequences have provided significant insights into human evolution and the genetic determinants of complex diseases and traits. Despite these advances, challenges persist in optimizing the integration of phasing tools, merging haplotype reference panels (HRPs), developing imputation algorithms, and fully exploiting the diverse applications of post-imputation data. This review comprehensively summarizes the advancements, applications, limitations, and future directions of HRPs in human genomics research. Recent progress in the reconstruction of HRPs, based on over 830,000 human whole-genome sequences, has been synthesized, highlighting the broad spectrum of human genetic diversity captured. Additionally, we recapitulate advancements in 56 HRPs for global and regional populations. The evaluation of imputation accuracy indicated that Beagle and Glimpse are the most effective tools for phasing and imputing data from genotyping arrays and low-coverage sequencing, respectively. A critical strategy for selecting an appropriate HRP involves matching the population background of target groups with HRP reference populations and considering multi-ancestry or homogeneous genetic structures. The necessity of a single, integrative, high-quality HRP that captures haplotype structures and genetic diversity across various genetic variation types from globally representative populations is emphasized to support both modern and ancient genomic research and advance human precision medicine.

## Graphical abstract

**Figure:**
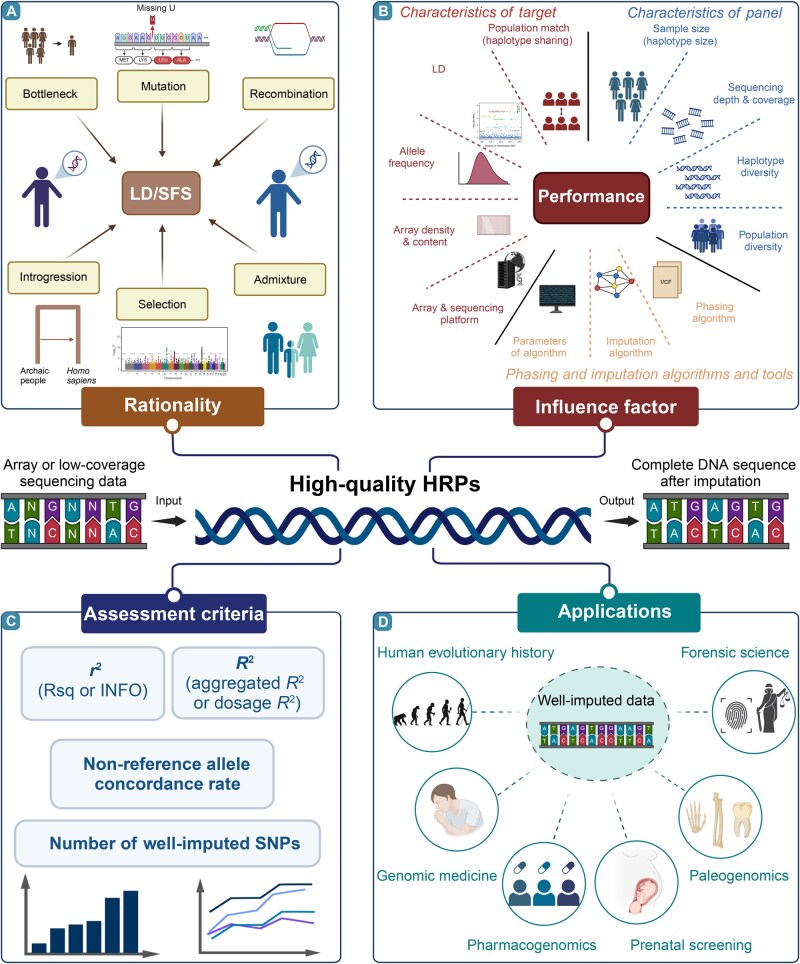

## Introduction

High-coverage whole-genome sequencing (WGS) has long been regarded as the gold standard for genotyping and identifying single-nucleotide polymorphisms (SNPs), insertions and deletions (InDels), and other structural variations (SVs) [1–6]. However, its high cost presents a significant barrier to population-scale genomic studies involving large cohorts. Consequently, much of the current research on modern humans depends on microarrays or low-depth WGS, which complicates genotype calling and restricts the ability to capture global genetic diversity [7]. Similar challenges have been observed in studies involving ancient DNA (aDNA) [8] and human leukocyte antigen (HLA) genes [9,10]. The established theoretical frameworks suggest that individuals within genetically close populations share population-specific long haplotype segments inherited from common ancestors. Accordingly, population-specific haplotype reference panels (HRPs), which consist of DNA sequences characterized by linkage disequilibrium (LD) and are tailored to document patterns of human genetic diversity, have been developed to facilitate the imputation of common, low-frequency, and rare genetic variations not directly genotyped in medical genome research or missing variants in aDNA studies.

Genetic variant information can be predicted or inferred through genotype imputation based on the documented patterns of haplotype structure in HRPs [11]. The accuracy of imputation depends mainly on the genetic architecture of the target populations (multi-ancestry heterogeneous background or genetically homogeneous structures) [12–14], algorithms such as the hidden Markov model (HMM) or deep learning employed by phasing and imputation tools [12–14], and properties of HRPs, such as marker density, the allele frequency spectrum, and the composition of reference populations [15–17]. Most imputation tools, including IMPUTE [18] and Markov Chain Haplotyping algorithm (MaCH) [19], leverage the HMM algorithm; however, updates to these tools have primarily targeted improvements in computational efficiency or memory requirements. In contrast, advances in HRPs, designed to capture the full breadth of human genetic diversity across distinct modern populations at scale, have significantly enhanced the quality of HRPs and imputation performance [20]. As a result, numerous HRPs have been constructed based on global genomic projects, such as the 1000 Genomes Project (1KGP), the Haplotype Reference Consortium (HRC) program, and Trans-Omics for Precision Medicine (TOPMed) [1–3]. More recently, population-specific HRPs, tailored to regional populations such as Han Chinese from the NyuWa Genome resource, Westlake BioBank for Chinese (WBBC), the Chinese Academy of Sciences Precision Medicine Initiative (CASPMI) project, and China Metabolic Analytics Project (ChinaMAP), Korean HRPs from the Northeast Asian Reference Database (NARD), or meta-Asian HRPs from the South and East Asian reference Database (SEAD), have improved the genetic discovery of these target populations [4,14,21–23]. Following imputation, low-coverage WGS or array-based databases enable the capture of more low-frequency or rare variations, increasing marker density, enhancing the statistical power of large-scale genome-wide association studies (GWASs) or meta-based work across different cohorts, and offering a cost-effective genotyping approach for downstream analyses, including polygenic risk score (PRS) estimation, genetic genealogy reconstruction, and demographic genetic history inference [24,25].

**Figure 1:**
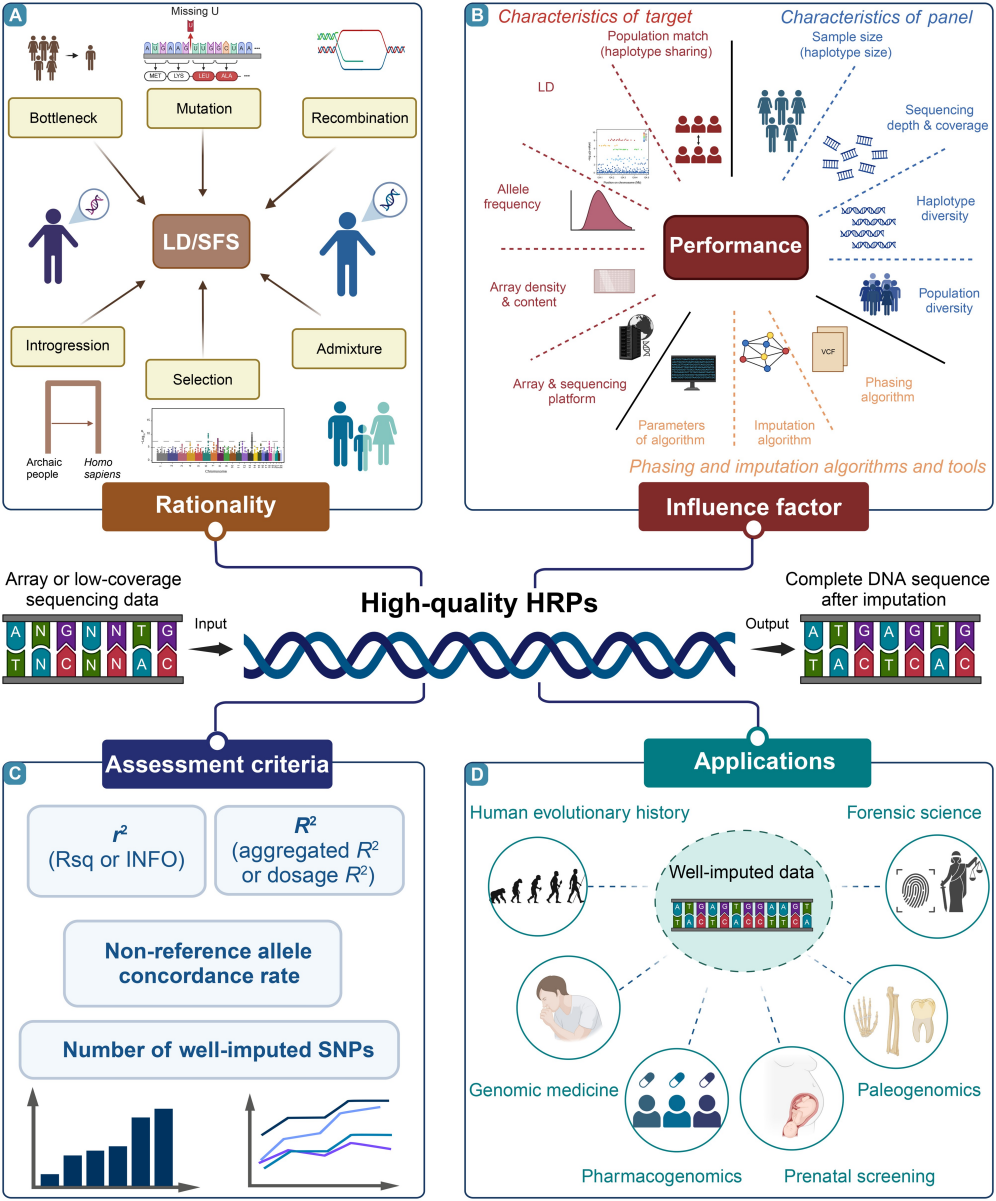
Summary of the HRP **A**. Differences in LD and SFS patterns among genetically distinct populations constitute the fundamental reason for the need for population-specific HRPs in precision medicine. These patterns are affected by multiple factors, including bottlenecks, mutations, admixture, introgression, recombination, and selection. **B**. Factors influencing the efficiency of HRPs include the following three aspects: the characteristics of the HRP (sample size, sequencing depth and coverage, haplotype diversity, and population diversity), the type and parameters of phasing and imputation tools, and the characteristics of the target population to be imputed (array or sequencing platform, array density and content, allele frequencies, LD patterns between loci, and the matching of the target population with the populations in the HRP). **C**. Metrics for measuring imputation quality include the statistics generated by imputation tools (*r*^2^ or INFO score), the squared Pearson’s correlation coefficients (aggregated *R*^2^ or dosage *R*^2^) between the true genotypes and imputed genotype dosages, non-reference allele concordance rate, and the number of well-imputed SNPs. **D**. Well-imputed data are applied in genomics studies of human evolutionary history, genomic medicine, pharmacogenomics, prenatal screening, paleogenomics, and forensic science. HRP, haplotype reference panel; LD, linkage disequilibrium; SFS, site frequency spectrum; SNP, single-nucleotide polymorphism.

The imputation process introduces systematic errors that are difficult to avoid, particularly when analyzing rare and low-frequency variants [26]. As the minor allele frequency (MAF) decreases, the imputation error rate increases [27]. Furthermore, imputation performance reliant on LD is shaped by haplotype patterns and variant spectra, which vary across genetically distinct populations [4]. Recent genetic studies have demonstrated that demographic events, including severe human bottlenecks occurring during migration out of Africa, along with biological adaptations driven by environmental extremes, pathogen exposure, dietary changes [28], and other evolutionary forces, such as mutation, migration, admixture, introgression, and recombination, significantly reshaped patterns of genetic diversity, allele frequency spectra, and LD patterns (**Figure 1**A). Template switching rates of theta values in the multi-ancestry HRPs also influence the imputation accuracy and imputation quality metrics of minor ancestry [29]. Populations with closer genetic affinities tend to share longer haplotypes and similar variant spectra, consistent with coalescence theory, which facilitates the acquisition of more precise haplotype information. However, HRPs, primarily established from European-specific haplotype data, have proven inadequate for genotype imputation in African, Asian, and other underrepresented indigenous populations [30,31]. This European-centric bias has led to inaccuracies in interpreting population-specific genetic foundations and conducting medical genetic studies focused on complex phenotypes in genetically diverse non-European populations [22,32,33]. Evidence indicates that reducing imputation error rates for low-coverage WGS and low-density array data can be achieved through factors such as larger sample sizes, deeper sequencing depths, better population representation, and closer genetic matching between reference and target populations (Figure 1B) [20,34,35].

Thus, constructing an integrative HRP encompassing worldwide population-specific haplotypes with diverse patterns of genetic diversity remains critical for advancing both population and medical genetics research. The foundational concepts and evaluation of HRPs have been extensively reviewed in prior studies [36,37]. Earlier reviews provide detailed insights into the interplay between imputation advancements and GWASs, particularly regarding common and rare diseases [11,36,38]. Over the past eight years, human genomics research and computational genomics have undergone transformative and explosive advancements driven by innovations in multi-ancestry and population-specific HRPs. These developments have significantly advanced both basic research and clinical applications [1,14,25,39,40]. This review synthesizes recent advances in HRPs and genotype imputation tools, providing an overview of five key areas: (1) the progress and geographical and ethnic distribution patterns of populations in global human genome projects and corresponding HRPs, with a focus on large-scale multi-ancestry or population-specific high-quality HRPs; (2) the current status and efficiency of state-of-the-art phasing and imputation tools in the era of large-scale genomics; (3) performance assessments of publicly available HRPs and the necessity for high-quality merged HRPs (Figure 1C); (4) applications of imputed datasets in human genetics, genome science, genome medicine, and forensic science (Figure 1D); and (5) challenges in HRP applications and future directions in the context of the telomere-to-telomere (T2T)/pangenome-based paradigm shift.

## Advances in worldwide human genome projects and corresponding HRPs in the past two decades

**Figure 2:**
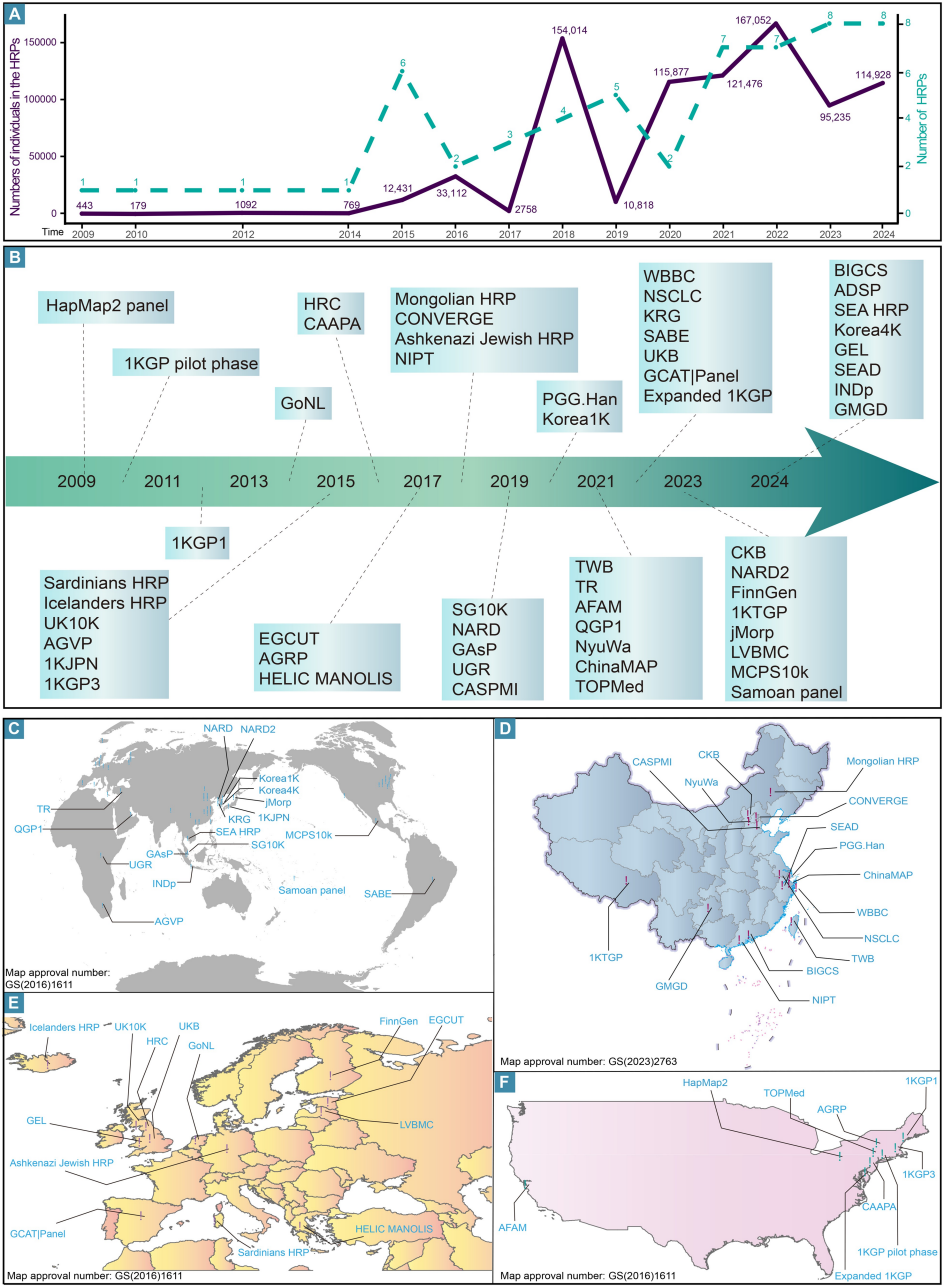
Geographical distribution and timeline of published HRPs **A**. Line charts showing the number of HRPs published annually (green dashed line) and their sample sizes (purple solid line). **B**. Timeline of published genome projects. **C**.–**F**. HRPs in different regions around the world (C), in China (D), in Europe (E), and in America (F). The annotations on the map represent only the general geographical location, and see Table S1 for details. HapMap, International HapMap Project; 1KGP, 1000 Genomes Project; GoNL, Genome of the Netherlands Project; UK10K, 10,000 UK Genome Sequences; AGVP, African Genome Variation Project; 1KJPN, a reference panel of 1070 Japanese individuals; HRC, Haplotype Reference Consortium (release 1.1); CAAPA, Consortium on Asthma among African-ancestry Populations in the Americas; HELIC MANOLIS, HELIC Pomak collection and the MANOLIS Cohorts; HELIC, Hellenic Isolated Cohorts; MANOLIS, Mylopotamos; EGCUT, Estonian Biobank of the Estonian Genome Center, University of Tartu; AGRP, Anabaptist Genome Reference Panel; NIPT, Non-invasive prenatal testing in China; CONVERGE, the China, Oxford and Virginia Commonwealth University Experimental Research on Genetic Epidemiology; SG10K, whole-genome-sequence 10,000 Singaporeans; NARD, Northeast Asian Reference Database; GAsP, GenomeAsia 100K Project pilot phase; UGR, Uganda Genome Resource; CASPMI, the Chinese Academy of Sciences Precision Medicine Initiative project; PGG.Han, Han Chinese Genome Initiative; Korea1K, the Korean Genome Project; TWB, Taiwan Biobank; TR, Turkish Variome; TOPMed, Trans-Omics for Precision Medicine; QGP1, Qatar Genome Program Phase 1; NyuWa, the NyuWa Genome resource; ChinaMAP, China Metabolic Analytics Project; AFAM, African Americans reference panel; WBBC, Westlake BioBank for Chinese pilot project; GCAT|Panel, GCAT|Genomes for Life Cohort; NSCLC, Non-small cell lung cancer in China; KRG, the Korean Reference Genome project; SABE, The Health, Well-being and Aging Study; CKB, China Kadoorie Biobank; UKB, UK Biobank; FinnGen, FinnGen project; 1KTGP, 1000 Tibetans Genomes Project; jMorp, The Tohoku Medical Megabank project; MCPS10k, the Mexico City Prospective Study; Samoan panel, Samoan-specific genotype reference panels; LVBMC, the Latvian population-specific reference panel; BIGCS, Born in Guangzhou Cohort Study; ADSP, the Alzheimer’s Disease Sequencing Project; SEA HRP, the Southeast Asian Specific Reference Panel; Korea4K, the Korean Genome Project; GEL, the Genomics England dataset; SEAD, the South and East Asian Reference Database reference panel; INDp, the Indonesian panel; GMGD, Guizhou Multi-Ethnic Genome Database.

The draft human genome sequence was completed two decades ago, marking a pivotal advancement supported by the Genome Reference Consortium’s publication of human reference genomes. Innovations in sequencing and computational techniques have accelerated the transition from single-genome studies to large-scale population genomic projects. In 2005, the International HapMap Consortium released the first haplotype map of the human genome, followed by the first HRP [20,41], marking a significant milestone (**Figure 2**A). Subsequent phases, including the pilot, phase 1, and phase 3 of the 1KGP, as well as the expanded 1KGP leveraging high-depth WGS technology, have provided valuable insights into the genomic diversity across major continental populations (Figure 2B) [3,42–44]. These initiatives have uncovered previously uncharacterized gaps in missing sequences and diversity, which were attributed to the limitations of array-based genotyping in earlier studies, and have also identified extensive human genetic variations, such as SNPs, InDels, and SVs. This progress heralded a new era of comprehensive human DNA sequencing and research. The availability of data from these genomic projects has enabled integrative analyses of human genome variations, contributing to the development of numerous large-size, multi-ancestry, high-quality HRPs or regional population-specific HRPs. These efforts have significantly advanced our understanding of the genomic architecture and genetic determinants of clinical diseases and complex physical traits (Figure 2C–F; **Table 1**, Table S1).

**Table 1:** Key characteristics and comparisons of all HRPs

Name	Published time	Sample size	Ancestry	Depth	Total number of variants	Phasing algorithm	Imputation algorithm	Assessment criterion	Target population	Imputation performance measurement
HapMap2 panel	2009	443	Multi-ethnic	/	513,008	/	MaCH	*r* ^2^	/	/
1KGP pilot phase	2010	179	Multi-ethnic	3.6×	16,224,519	/	IMPUTE	*r* ^2^	/	/
1KGP1	2012	1092	Multi-ethnic	5.1×	28,975,367	SHAPEIT2	Beagle	*R* ^2^	/	SHAPEIT2 *vs*. Thunder *vs*. Beagle
GoNL	2014	769	Netherlands	13×	21,524,538	BEAGLE	IMPUTE2	*R* ^2^	Dutch	GoNL + 1KGP *vs*. GoNL + 1KGP1 European *vs*. GoNL *vs*. 1KGP1 *vs*. 1KGP1 European
Sardinians HRP	2015	2120	Sardinians	20×	21,131,214	/	/	*r* ^2^	Sardinians	Sardinians HRP *vs*. 1KGP phase3 *vs*. 1KGP phase1
Icelanders HRP	2015	2636	Iceland	7×	45,492,035	SHAPEIT2	Beagle	*R* ^2^	/	/
UK10K	2015	3781	European	4×	29,809,603	SHAPEIT2	IMPUTE2	*r* ^2^	UK	UK10K + 1KGP *vs*. UK10K *vs*. 1KGP
AGVP	2015	320	African	32.4×	24,574,727	SHAPEIT2	/	*r* ^2^	/	/
1KJPN	2015	1070	Japanese	7.4×	49,143,605	SHAPEIT2	IMPUTE2	*R* ^2^	Japanese	1KJPN + 1KGP *vs*. 1KJPN *vs*. 1KGP *vs*. 1KGP JPT
1KGP3	2015	2504	Multi-ethnic	4×	17,600,000	MaCH	Minimac	*R* ^2^	Multi-ethnic	1KGP phase3 *vs*. 1KGP phase1
HRC	2016	32,470	European	4×–8×	39,635,008	SHAPEIT3	IMPUTE2	*R* ^2^	CEU	HRC *vs*. UK10K *vs*. 1KGP3
CAAPA	2016	642	African	35×	41,163,897	/	/	/	/	/
HELIC MANOLIS	2017	249	Cretan	4×	9,554,503	SHAPEIT2	IMPUTE2	*r* ^2^	/	/
EGCUT	2017	2244	Estonian	30×	16,536,512	SHAPEIT2	IMPUTE2	r^2^/number of well-imputed SNVs	/	/
AGRP	2017	265	Amish and Mennonite	30×	1,081,253	SHAPEIT2	IMPUTE2	*R* ^2^	AGRP	AGRP + 1KGP *vs*. HRC *vs*. AGRP *vs*. 1KGP
NIPT	2018	141,431	Chinese	0.06×	9,040,000	/	STITCH	*r* ^2^/number of well-imputed SNVs	/	/
Mongolian HRP	2018	175	Mongolian	21.8×	16,526,134	SHAPEIT2	IMPUTE2	*R* ^2^	Mongolian	Mongolian HRP + 1KGP3 *vs*. Mongolian HRP *vs*. 1KGP3 East Asian + Mongolian HRP *vs*. 1KGP3
AJ HRP	2018	738	Ashkenazi Jewish	30×	26,400,000	SHAPEIT2	IMPUTE2	*R* ^2^/genotype discordance	Ashkenazi Jewish	AJ HRP + 1KGP3 *vs*. AJ HRP *vs*. HRC *vs*. 1KGP3 *vs*. UK10K + 1KGP *vs*. UK10K *vs*. 1KGP
CONVERGE	2018	11,670	Chinese	1.7×	24,114,249	/	/	/	/	/
SG10K	2019	4810	Multi-ethnic	13.7×	98,273,706	Eagle2	Beagle4	*r* ^2^	Multi-ethnic	SG10K + 1KGP3 *vs*. SG10K *vs*. 1KGP3
NARD	2019	1779	Multi-ethnic	10×–20×	44,444,122	SHAPEIT3	Minimac3	*R* ^2^/genotype discordance	Korean	NARD + 1KGP3 *vs*. NARD *vs*. 1KGP3 *vs*. HRC
GAsP	2019	1654	Asian	30×	21,494,814	SHAPEIT2	Eagle2	/	/	/
UGR	2019	1978	African	4×	46,000,000	SHAPEIT2	IMPUTE2	/	/	/
CASPMI	2019	597	Chinese	25×–35×	/	/	/	/	/	/
PGG.Han	2020	114,783	Chinese	30×–80×	8,056,973	/	/	/	/	/
Korea1K	2020	1094	Korean	31×	38,800,000	SHAPEIT2	Minimac3	R^2^	Korean	Korea1K + 1KGP3 *vs*. Korea1K *vs*. 1KGP3
TWB	2021	1445	Chinese	/	/	SHAPEIT2	SHAPEIT2	*R* ^2^/concordance	Chinese	TWB + 1KGP East Asian *vs*. TWB *vs*. 1KGP East Asian
TR	2021	773	Turkish	34×	45,981,721	SHAPEIT2	IMPUTE2	*R* ^2^	Balkan	TR + 1KGP3 *vs*. TR *vs*. 1KGP3
TOPMed	2021	97,256	Multi-ethnic	30×	308,107,085	Eagle2	Minimac4	*R* ^2^	UKB	/
QGP1	2021	6218	Qatari	30×	68,107,887	Eagle2	Minimac3	*R* ^2^/number of well-imputed SNVs/*r*^2^	Qatari	QGP *vs*. 1KGP3 *vs*. HRC *vs*. CAAPA *vs*. HapMap2
NyuWa	2021	2902	Chinese	26.2×	19,256,267	SHAPEIT4	Minimac4	*R* ^2^	Multi-ethnic	NyuWa + 1KGP3 *vs*. NyuWa *vs*. TOPMed *vs*. GAsP *vs*. 1KGP3 *vs*. HRC
ChinaMAP	2021	10,588	Chinese	40.8×	59,010,860	/	/	/	/	/
AFAM	2021	2294	Sub-Saharan African	15×	52,500,000	SHAPEIT4	Minimac4	*R* ^2^	African Americans	TOPMed *vs*. AFAM *vs*. 1KGP3 *vs*. HRC *vs*. CAAPA
WBBC	2022	4535	Chinese	13.9×	81,498,995	SHAPEIT2	Minimac4	r^2^/number of well-imputed SNVs/non-reference genotype concordance rate	Multi-ethnic	WBBC + 1KGP East Asian *vs*. WBBC + 1KGP *vs*. WBBC *vs*. 1KGP3 *vs*. CONVERGE
UKB	2022	149,960	Multi-ethnic	32.5×	643,747,446	/	IMPUTE2	/	/	/
NSCLC	2022	6004	Chinese	30×	100,565,590	SHAPEIT4	Minimac4	*r* ^2^/number of well-imputed SNVs	Chinese	NSCLC *vs*. 1KGP3
GCAT|Panel	2022	690	European	30×	35,431,441	SHAPEIT4	IMPUTE2	*r* ^2^	Multi-ethnic	/
Expanded 1KGP	2022	3202	Multi-ethnic	34×	70,768,225	SHAPEIT2	IMPUTE2	*R* ^2^	Multi-ethnic	/
KRG pilot	2022	1490	Korean	29.0×	13,637,761	SHAPEIT4	Minimac4	*R* ^2^/number of well-imputed SNVs	Multi-ethnic	Differences across target populations
SABE	2022	1171	Brazilians	38.6×	/	SHAPEIT2	IMPUTE2	*r* ^2^	Brazilians	SABE + 1KGP3 *vs*. SABE *vs*. 1KGP3
CKB	2023	9964	Chinese	15.41×	129,743,542	Beagle5.2	Beagle5.2	*R* ^2^/number of well-imputed SNVs/precision/sensitivity	Chinese	ChinaMAP *vs*. CKB *vs*. TOPMed *vs*. NyuWa *vs*. extended 1KGP
NARD2	2023	14,393	Multi-ethnic	/	/	Beagle5.0	Minimac4	*R* ^2^/number of well-imputed SNVs	KOR	NARD2 *vs*. NARD *vs*. TOPMed
FinnGen	2023	3775	Finnish	30×	/	Beagle4.1	Beagle4.1	*r* ^2^	/	/
1KTGP	2023	1064	Chinese	11.8×	28,200,000	SHAPEIT2	IMPUTE2	/	/	/
jMorp	2023	54,302	Japanese	/	/	/	/	/	/	/
MCPS10k	2023	9950	Mexican	30×	/	SHAPEIT4	IMPUTE5	*R* ^2^	MCPS individuals	MCPS10k *vs*. TOPMed
Samoan panel	2023	1285	Samoan	/	/	Eagle2	Minimac4	*r* ^2^/number of well-imputed SNVs	Samoans	1KGP3 + Samoan panel *vs*. TOPMed *vs*. 1KGP3
LVBMC	2023	502	Latvian	35.7×	/	Eagle2	Beagle4.1	Number of well-imputed SNVs	Latvians	/
BIGCS	2024	2245	Chinese	6.63×	47,239,473	Beagle4.0	Minimac3	*R* ^2^	Chinese	BIGCS + TOPMed *vs*. BIGCS *vs*. TOPMed *vs*. 1KGP3 *vs*. GAsP *vs*. HRC
ADSP	2024	16,564	Multi-ethnic	/	54,000,000	SHAPEIT4	Minimac3	*R* ^2^/*r*^2^	Multi-ethnic	Differences across target populations
SEA HRP	2024	2550	Southeast Asian	/	113,851,450	Beagle5 *vs*. SHAPEIT4 and IMPUTE5		*r* ^2^/number of well-imputed SNVs/non-reference disconcordance rate	Orang Asli	SEA HRP *vs*. 1KGP3
Korea4K	2024	3614	Korean	27.75×	26,210,741	SHAPEIT2	Minimac3	*R* ^2^	Korean	Korea4K *vs*. Korea1K
GEL	2024	78,195	European	30×	342,573,817	SHAPEIT4	IMPUTE5	*r* ^2^	Multi-ethnic	Differences across target populations
SEAD	2024	11,067	Multi-ethnic	/	80,367,720	SHAPEIT2	Minimac4	*r* ^2^/number of well-imputed SNVs/non-reference concordance rate	Chinese	SEAD *vs*. SG10K *vs*. WBBC *vs*. 1KGP *vs*. GAsP
INDp	2024	217	Indonesian	30×	10,144,296	SHAPEIT2	IMPUTE2	*r* ^2^/non-reference concordance rate	West Javanese	INDp + 1KGP East Asian *vs*. INDp *vs*. 1KGP East Asian
GMGD	2024	476	Chinese	5.5×	16,336,982	Beagle5.2	Beagle5.2	*R* ^2^	/	/

*Note*: *R*^2^, the squared Pearson’s correlation coefficient (aggregated *R*^2^ or dosage *R*^2^) between the true genotypes and imputed genotype dosages. *r*^2^, the score generated by software without the true genotype (Rsq and INFO). In the “imputation performance measurement” column, we list the reference panels in order of their performance in the target population, from best to worst. The complete details can be found in Table S1. HRP, haplotype reference panel; MaCH, Markov Chain Haplotyping algorithm; HapMap, International HapMap Project; 1KGP, 1000 Genomes Project; GoNL, Genome of the Netherlands Project; UK10K, 10,000 UK Genome Sequences; AGVP, African Genome Variation Project; 1KJPN, a reference panel of 1070 Japanese individuals; HRC, Haplotype Reference Consortium (release 1.1); CAAPA, Consortium on Asthma among African-ancestry Populations in the Americas; HELIC MANOLIS, HELIC Pomak collection and the MANOLIS Cohorts; HELIC, Hellenic Isolated Cohorts; MANOLIS, Mylopotamos; EGCUT, Estonian Biobank of the Estonian Genome Center, University of Tartu; AGRP, Anabaptist Genome Reference Panel; NIPT, Non-invasive prenatal testing in China; CONVERGE, the China, Oxford and Virginia Commonwealth University Experimental Research on Genetic Epidemiology; SG10K, whole-genome-sequence 10,000 Singaporeans; NARD, Northeast Asian Reference Database; GAsP, GenomeAsia 100K Project pilot phase; UGR, Uganda Genome Resource; CASPMI, the Chinese Academy of Sciences Precision Medicine Initiative project; PGG.Han, Han Chinese Genome Initiative; Korea1K, the Korean Genome Project; TWB, Taiwan Biobank; TR, Turkish Variome; TOPMed, Trans-Omics for Precision Medicine; QGP1, Qatar Genome Program Phase 1; NyuWa, the NyuWa Genome resource; ChinaMAP, China Metabolic Analytics Project; AFAM, African Americans reference panel; WBBC, Westlake BioBank for Chinese pilot project; GCAT|Panel, GCAT|Genomes for Life Cohort; NSCLC, Non-small cell lung cancer in China; KRG, the Korean Reference Genome project; SABE, The Health, Well-being and Aging Study; CKB, China Kadoorie Biobank; UKB, UK Biobank; FinnGen, FinnGen project; 1KTGP, 1000 Tibetans Genomes Project; jMorp, The Tohoku Medical Megabank project; MCPS10k, the Mexico City Prospective Study; Samoan panel, Samoan-specific genotype reference panels; LVBMC, the Latvian population-specific reference panel; BIGCS, Born in Guangzhou Cohort Study; ADSP, the Alzheimer’s Disease Sequencing Project; SEA HRP, the Southeast Asian Specific Reference Panel; Korea4K, the Korean Genome Project; GEL, the Genomics England dataset; SEAD, the South and East Asian Reference Database reference panel; INDp, the Indonesian panel; GMGD, Guizhou Multi-Ethnic Genome Database; JPT, Japanese in Tokyo, Japan; CEU, Utah Residents with Northern and Western European Ancestry; SNV, single-nucleotide variant.

Recent advancements in high-quality HRP innovations have emerged at a rapid pace. In Europe, the HRC program and subsequent European genomic initiatives, including the Genome of the Netherlands Project (GoNL), 10,000 UK Genome Sequences (UK10K), Genomics England (GEL), and other genomic studies of Estonians, Sardinians, Cretans, Ashkenazi Jewish, Icelanders, and Latvians, have yielded high-quality HRPs and extensive genetic datasets [39,45–58]. These large-scale human genomic resources have facilitated high-quality HRP construction, fine-scale genetic analyses, and significant advancements in precision medicine across diverse European populations (Figure 2E). Genetic analysis revealed a striking European bias in human genome research, with 86% of GWASs conducted in populations of European ancestry, resulting in an overrepresentation of European populations in HRPs [28,33,59]. Efforts to develop genomic projects focused on Asian populations have aimed to address the underrepresentation and selection bias of non-European groups in human genome research and to illuminate novel genetic determinants of East Asian-specific diseases and health traits. Over 30 initiatives focused on East Asians, Southeast Asians, or single-ethnic groups such as Chinese Mongolians and Tibetans have recently been launched, significantly contributing to this effort (Figure 2C) [4,12,13,22,23,32,60–82]. Northern Americans, historically dominated by migrants from other continents with complex mixed ancestry, exhibit diverse genetic backgrounds. The peopling of the Americas is traced back to at least the late Paleolithic period [83,84]. Native American populations possess 14% to 38% ancestry related to MA-1, a 24,000-year-old individual from Mal’ta in south-central Siberia, with the remainder of their ancestry deriving from East Asians [83,84]. Latin American populations comprise both admixed and predominantly indigenous subpopulations, many of which remain genetically uncharacterized. Genomic initiatives in Northern America, such as the TOPMed program, the Consortium on Asthma among African-ancestry Populations in the Americas (CAAPA), the African Americans reference panel (AFAM), the Anabaptist Genome Reference Panel (AGRP), and others, have aimed to diversify genetic collections across these groups (Figure 2F) [1,40,85–90]. Africa, recognized as the cradle of modern humans, harbors immense genetic and linguistic diversity, including Afro-Asiatic, Nilo-Saharan, Khoisan, and Niger-Congo language groups. However, few cohort studies have been conducted to explore the genetic architecture and disease susceptibility in African populations, with notable exceptions including the African Genome Variation Project and Uganda Genome Resource [31,91]. For Pacific islanders, only one HRP specific to Samoan haplotype genotypes has been developed, while public reference panels lack sufficient samples from Oceania. This underrepresentation exacerbates the health disparities faced by Pacific islanders [92]. Collectively, these genomic projects and the reference panels developed in parallel underscore global efforts to advance equitable healthcare and precision medicine. Seventeen of these panels are accessible through publicly available online imputation platforms, encompassing samples from European, Asian, African, and indigenous Oceanian ancestries (Table S1).

**Figure 3:**
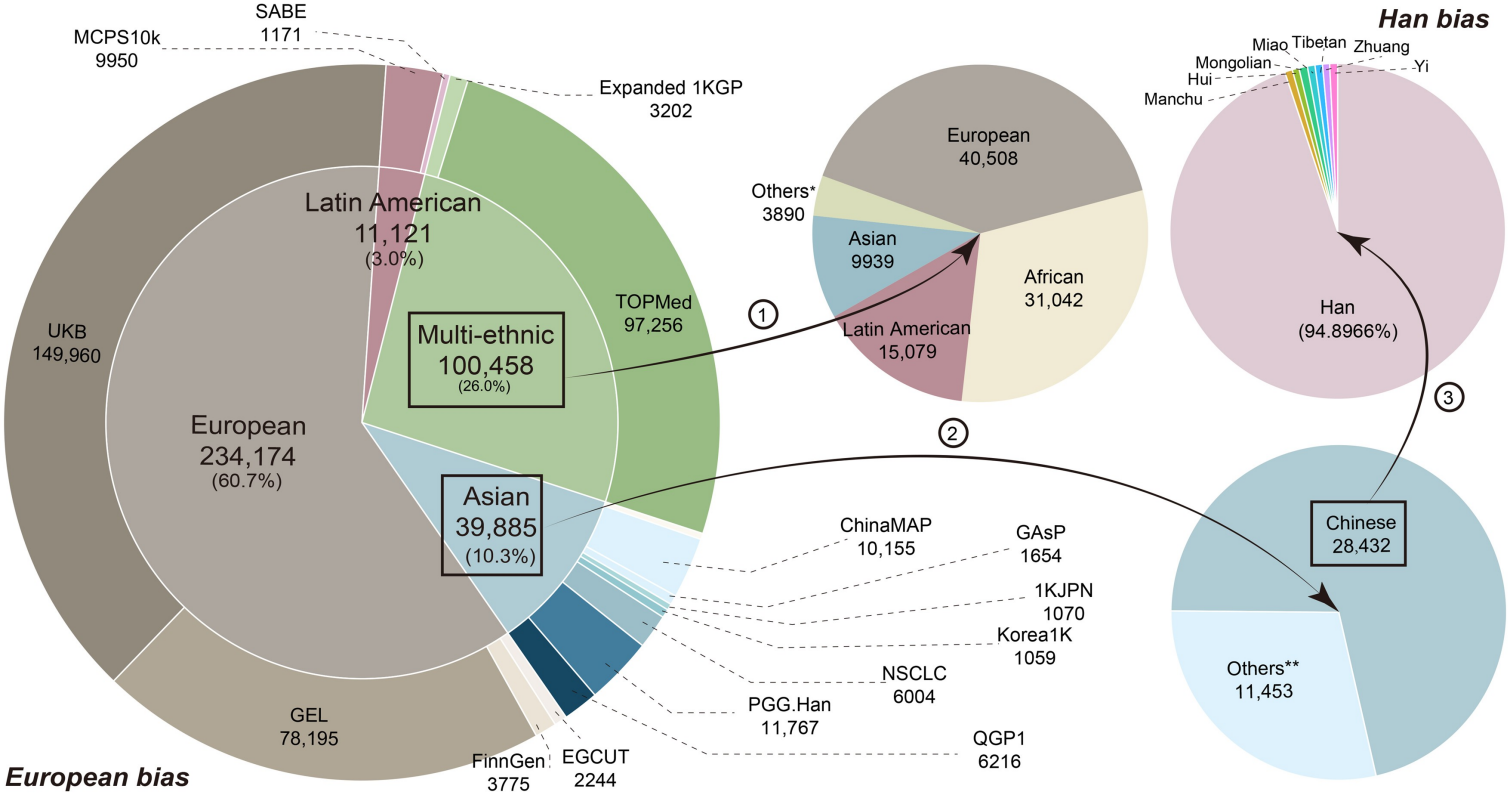
Number of samples and proportion of ancestry in the high-quality HRPs The high-quality HRPs have a sequencing depth of ≥ 30× and include more than 1000 individuals. In the left pie chart, the inner ring represents the distribution of samples by ancestral composition, whereas the outer ring reflects the distribution of HRPs. Importantly, the presence of duplicate individuals in the integrated reference panel was not excluded. Others* includes Samoan, Native American, multiple groups, or unknown ancestries. Others** includes Indian, Malaysian, South Korean, Pakistani, Mongolian, Papuan New Guinean, Indonesian, Philippine, Japanese, and Russian.

In addition to enhancing diversity by including genetically distinct continental populations, imputation accuracy was found to improve with an increased number of haplotypes and samples in the HRPs, particularly for rare and low-frequency variants [11,25,38]. Additionally, closer genetic proximity between the target population and the populations in HRPs corresponded to higher imputation accuracy, as demonstrated in these validation studies [25,58,93,94]. For example, when the TOPMed HRP was used, the mean imputation *r*-squared (*r*^2^) for European variants was 0.93, compared to 0.62 for Papua New Guineans [25]. For populations with excess African ancestry, both the TOPMed and AGRP HRPs demonstrated superior imputation accuracy [95]. Meta-imputation utilizing HRPs from the TOPMed and the expanded 1KGP yielded the best results for the Pakistani population [96]. The inclusion of diverse reference panels has been shown to increase imputation accuracy for rare variants significantly [38]. Analysis of sample size and ancestry composition in high-quality HRPs (sequencing depth ≥ 30×, sample size > 1000) revealed that individuals of European ancestry accounted for 60.7%. In fine-scale regional or isolated populations, the selection bias or Han bias has hindered the equitable representation of ethnolinguistically diverse ethnic minorities within regional population genomic cohorts (**Figure 3**). Asian HRPs with unspecified sequencing depths were excluded from this analysis (Table S1). While large-scale, population-specific initiatives are essential for unlocking the full potential of genome sequencing, non-European populations remain underrepresented in genomic studies owing to the high cost of sequencing. Consequently, the development of high-quality, population-specific HRPs may offer a more effective strategy for advancing genomic research in developing countries. Given the limited integration of population genetic backgrounds in current molecular anthropological and medical studies, a combined approach is recommended. This approach would involve merging high-quality sequencing data from genetically diverse cohorts, such as the 1KGP and Human Genome Diversity Project (HGDP), or integrating population-specific reference panels with multi-ethnic resources, such as the TOPMed and SEAD reference panels. Such efforts would facilitate the creation of a comprehensive, multi-ancestry, high-quality HRP that better represents underrepresented populations or those with mixed ancestral origins, including European, American, Oceanian, Singaporean, and South African populations.

## Optimal combination of phasing and imputation tools

Haplotype phasing, including methods such as family-based phasing, physical phasing, and LD-based phasing, serves as a cornerstone in statistical and population genetics. This approach facilitates the identification of genetic variant combinations on individual chromosomes and underpins the construction of reference panels. Family-based phasing utilizes kinship information, including trios or multigenerational family data, to allocate alleles to haplotypes with high accuracy based on Mendelian inheritance patterns. However, its efficacy relies on the availability of comprehensive family datasets. Physical phasing employs experimental approaches, such as long-read and single-molecule sequencing technologies (*e.g.*, PacBio and Nanopore) and next-generation sequencing, to determine haplotypes directly, often leveraging trios or physically dissected chromosomal markers. LD-based phasing infers haplotypes via LD patterns derived from population-level data. Tools such as SHAPEIT [97] and Beagle [98] have been developed to facilitate haplotype phasing in individuals without family data. However, the accuracy of these methods can be affected by population-specific LD structures.

In the current well-designed statistical phasing strategy applied to array-based data, whole-genome data [whole-exome sequencing (WES) and WGS] primarily rely on haplotype diversity patterns driven by LD within populations or utilize coalescent theory to identify shared identity-by-descent (IBD) segments among samples [99]. The most widely used phasing tools include Eagle [17], SHAPEIT [97], and Beagle [98] (Table S2). Phasing performance is typically assessed using trio samples to establish the true haplotypes of offspring, which are then compared with those inferred by phasing algorithms. Phasing accuracy was evaluated through the switch error rate (SER), defined as the fraction of consecutive heterozygous genotypes that are incorrectly phased. In an analysis of array data from 500 European trio samples, the reported SERs were as follows: SHAPEIT4 (0.117%), Beagle5 (0.125%), and Eagle2 (0.178%) [100]. Updates to SHAPEIT5 and Beagle5.4 have further improved the phasing accuracy for rare variants. In evaluations with European trio samples, both methods demonstrated low SERs (< 0.2%) when considering Axiom array loci alone. However, for rare variants in WGS data (with minor allele counts between 11 and 20 or allele frequencies less than 0.01), their SERs were 4.36% and 8.76%, respectively, whereas Eagle2 did not significantly improve the phasing performance for rare variants. Enhanced phasing accuracy for rare variants has also contributed to improved genotype imputation accuracy within corresponding high-resolution panels [97]. An independent assessment by Cole et al. examined the phasing performance of SHAPEIT5.1.1 and Beagle5.4 on genetically distinct populations (Table S2). Overall, Beagle demonstrated a lower SER than that SHAPEIT did, achieving extremely low SERs ranging from 0.012% to 0.028% for array data from individuals of European, Ashkenazi Jewish, African-admixed, and American-admixed ancestry. Conversely, average SERs for East Asian and South Asian individuals were reported to be 0.36% (standard deviation 0.17%) and 0.50% (standard deviation 0.34%), respectively. In WGS data, both SHAPEIT and Beagle displayed strong performance within European samples (SER = 0.021%) but exhibited suboptimal results for East Asian samples (SER = 3.49%) [101]. Under comparable conditions, phasing with Beagle yielded superior imputation results, and phasing with a reference panel outperformed reference-free approaches [102].

The imputation tools, developed to optimize uncertain genotype likelihoods and bridge gaps in sparsely mapped reads, were designed to maximize the utility of HRPs. Their application significantly improved the accuracy and statistical power of low-coverage WGS data following genotype imputation [39,103]. Constantly updated HRPs, incorporating more individuals from different ancestral populations and newly reported variants, facilitate iterative improvements to these tools, enhancing the accuracy of rare variant detection while minimizing computational time and memory requirements. Various genotyping and sequencing methods, including SNP tagging approaches for genotype-wide SNPs and low-coverage WGS, are commonly employed. Key tools in this domain include IMPUTE, Beagle, and Minimac (Table S2) [18,98,104]. Validation studies indicated that, under consistent conditions, the performance of these imputation tools was broadly comparable, with *R*^2^ differences of no more than 0.01. Beagle demonstrated superior performance, followed by IMPUTE, with Minimac ranking last [102]. Additionally, QUILT and Glimpse, which utilize Gibbs sampling and HMM, were designed explicitly for low-pass WGS data [105,106]. For low-coverage aDNA and non-invasive prenatal testing data, Glimpse slightly outperformed QUILT and Beagle [107,108]. Moreover, both versions of Glimpse exhibited similar accuracy levels for ancient genomic data [109,110], with version 1 showing superior accuracy for data with 0.1× coverage and variants with a MAF > 0.02. In conclusion, Beagle is recommended for phasing and imputation of array data using a unified HRP, while Glimpse remains the preferred tool for low-coverage WGS data in modern and ancient populations.

## Performance and disparities of diverse HRPs

The size and number of high-quality HRPs have increased with advancements in large-scale human genomic cohorts, necessitating the development of a comprehensive reference panel for global or regional population use. This is essential for the medical and population genetics communities. We systematically summarized the effects of multiple factors, such as the target population, reference panel composition, and computational strategies, on imputation accuracy (Figure 1). Recently, several HRPs have been validated as highly effective for genetically similar populations. However, limitations in publicly available resources and challenges in selecting appropriate panels for specific target populations have impeded their broader application. The genotype imputation accuracy was evaluated via two main metrics: *r*^2^, derived without reference to true genotypes, and aggregated *R*^2^ (also known as dosage *R*^2^), which reflects the squared Pearson’s correlation coefficients between imputed dosage and true genotypes [111]. Aggregated *R*^2^ values were obtained by grouping markers according to the MAF. In addition to standard indices, concordance between predicted and observed genotypes, high-*r*^2^ variants, and the density or coverage of high-quality imputed markers were incorporated as key metrics for evaluating the validation performance of the custom panels [25]. Bai et al. designed hundreds of customized reference panels with varying haplotype sizes and diversity to investigate the relationship between imputation accuracy and panel composition [37]. It was demonstrated that simulated reference panels with differing diversity yielded varying imputation performance in Han Chinese and European populations. For Han Chinese, imputation accuracy plateaued when haplotype diversity within the reference panels was limited. An intriguing explanation was proposed via this work, attributing higher “diversity acceptability” in Western Eurasians, which was inconsistent with contributions from three ancestral sources: European hunter-gatherers, Near East farmers, and Steppe Yamnaya herders [112]. This finding indicates that ancient complex demographic events have an obvious influence on imputation performance. This systematic evaluation underscores the need for large-scale, high-quality reference panels representing underrepresented populations, such as Han Chinese. To address this gap, the Han Chinese-specific WBBC panel was developed and integrated with multi-ancestry sources to form the SEAD panel, enhancing imputation accuracy for Han Chinese and broader Asian populations [14,63].

Cahoon et al. evaluated the imputation performance of the large-scale, multi-ancestry, state-of-the-art TOPMed reference panel, which demonstrated higher simulation accuracy in European populations but did not yield similar improvements for underrepresented non-European populations. The *r*^2^ estimates were shown to correlate with genetic distance to European populations and were overestimated in non-European populations [25]. Shi et al. further highlighted that estimated template switching rates in the meta-ancestry reference panel may contribute to inflated *r*^2^ values [29]. However, this had a limited impact on evaluating the imputation performance of population-specific panels. Comparative analyses have been conducted via genomics-based reference panels from various population cohorts. Notable examples include TOPMed and AGRP in Africans [95], ChinaMAP and WBBC for Han Chinese [13,63], and NARD for Northeast Asians [22]. These findings highlight the importance of incorporating diverse genomic resources to improve imputation precision and health equity.

**Figure 4:**
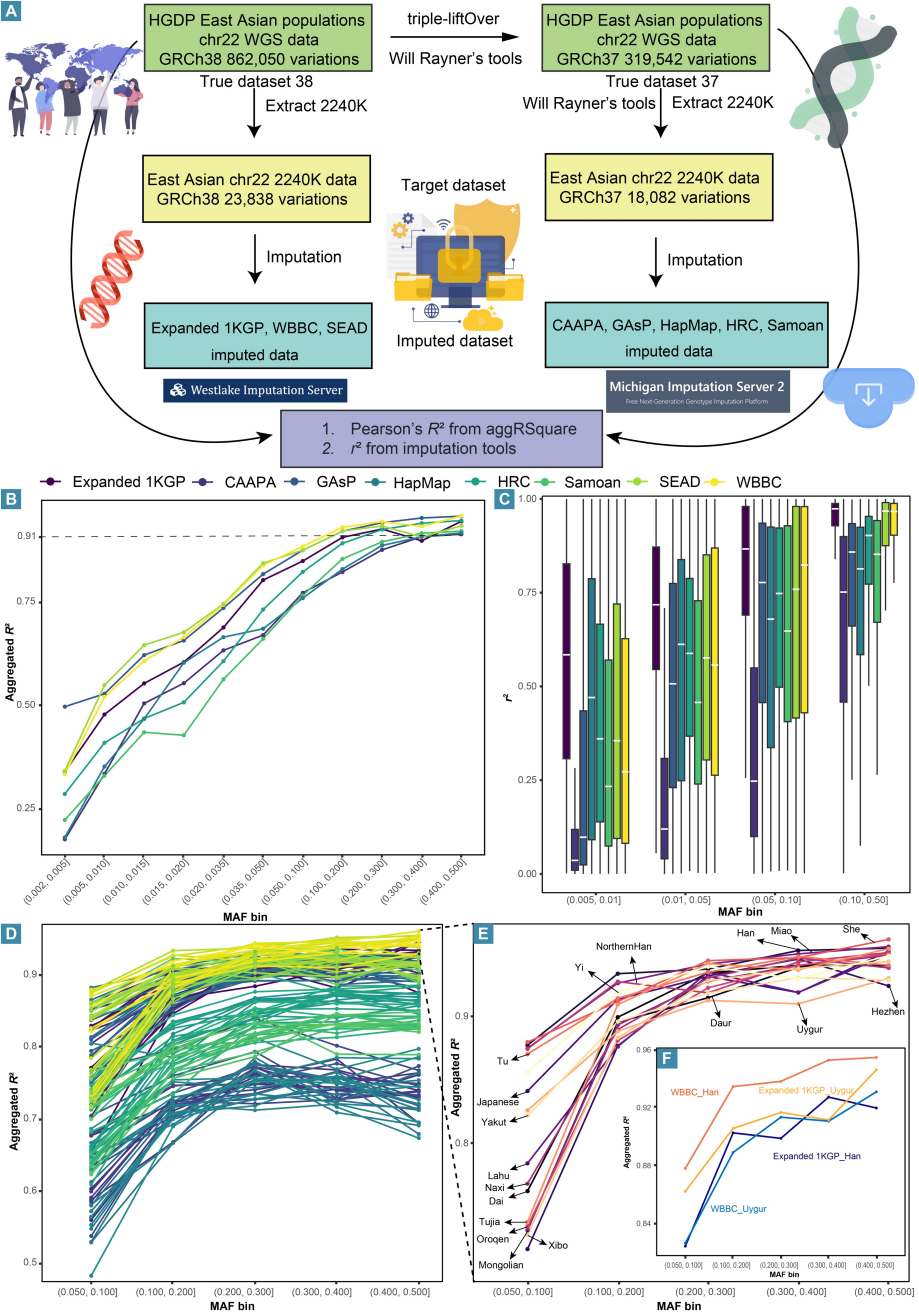
Evaluation of the performance of different reference panels **A**. Workflow of the evaluation process. **B**. Line chart showing the aggregated *R*^2^ values of the 8 HRPs across different MAF bins. Different colored lines represent different HRPs. A higher aggregated *R*^2^ indicates greater similarity between the imputed data and the true data. The dashed line indicates the threshold for high-quality imputation (aggregated *R*² = 0.91). **C**. Box plot showing the *r*^2^ values of the 8 HRPs across different MAF bins. Different colored boxes represent different HRPs. The *r*^2^ values are derived from the imputation tool’s evaluation without the true genotype. **D**. Imputation performance of 18 East Asian populations from HGDP across 8 HRPs. Each line represents a population, and lines with the same color correspond to the same HRP. **E**. Imputation performance of 18 East Asian populations from HGDP using the WBBC HRP. Different colored lines represent different populations. **F**. Imputation performance of the Han and Uyghur populations using the WBBC HRP (population-specific) and the expanded 1KGP HRP (multi-ancestry). WGS, whole-genome sequencing; HGDP, Human Genome Diversity Project; MAF, minor allele frequency.

We addressed this issue by imputing Chinese genomic diversity using all available high-quality HRPs. Our validation work underscored the importance of using population-matched reference panels for accurate genomic imputation. The imputation performance of publicly available HRPs was assessed via WGS data from 224 East Asian samples in the HGDP as the ground truth dataset. Quality control was conducted using VCFtools with parameters including “--maf 0.002”, “--max-missing 0.95”, “--minQ 30”, and “--hwe 1e-10”. Inconsistencies in reference genome versions among different HRPs were resolved using triple-liftOver to convert genomic coordinates to hg19 [113], and allele switches or strand flips were corrected by comparing allelic data against HRC data via Will Rayner’s tools. Imputation was halted if more than 10% of allele switches were detected during quality control (**Figure 4**A). Seventeen HRPs were accessed via imputation websites (Table S1), with imputed data obtained from eight usable HRPs after excluding HGDP samples already represented in the HRPs (**Table 2**).

**Table 2:** Basic information of publicly available HRPs and the number of variants obtained after imputing chromosome 22

Reference panel	Sample size	Reference genome	Ancestry	Number of imputed sites
Expanded 1KGP	3202	GRCh38	Multi-ethnic	680,128
CAAPA	642	GRCh37	African American	381,379
GAsP	1654	GRCh37	Asian	288,682
HRCr1.1	32,470	GRCh37	European	524,544
Samoan	1285	GRCh37	Oceanian	222,128
HapMap2	443	GRCh37	Multi-ethnic	33,805
WBBC	4535	GRCh38	Chinese	494,305
SEAD	11,067	GRCh38	Asian	1,228,248

*Note*: Expanded 1KGP, CAAPA, GAsP, HRCr1.1, Samoan, and HapMap2 were imputed at https://imputationserver.sph.umich.edu/#!pages/home, which uses Eagle2.4 and Minimac4. WBBC and SEAD were imputed at https://imputationserver.westlake.edu.cn/index.html via SHAPEIT2 and Minimac4.

The squared Pearson’s correlation coefficients (aggregated *R*^2^) between the true genotypes and imputed genotype dosages were calculated to assess imputation accuracy. The results demonstrated a positive correlation between imputation accuracy and increasing MAF. For variants with MAFs ranging from 0.002 to 0.005, the GAsP HRP exhibited the highest accuracy (aggregated *R*^2^ = 0.497). For variants with MAFs between 0.01 and 0.05, panels containing larger Asian sample sizes performed better, with SEAD achieving the highest accuracy (Figure 4B). Overall, the SEAD HRP was found to be the most suitable for the East Asian population in the HGDP. When *r*^2^ statistics were evaluated, the expanded 1KGP HRP showed overall advantages (Figure 4C), underscoring the potential for imputation tool metrics to overestimate accuracy and lead to erroneous conclusions [25,29]. Benchmarking should thus avoid relying solely on *r*^2^ or aggregated *R*^2^ values. Further evaluation of imputation performance across 18 East Asian populations indicated that the WBBC panel exhibited the highest overall performance, although imputation accuracy varied across populations (Figure 4D). The WBBC panel showed better performance for Han populations but poorer results for Uyghur population (Figure 4E), likely due to the predominance of Han samples in the panel, emphasizing the importance of population matching in enhancing imputation accuracy. In contrast, the Uyghur population achieved better accuracy with the more diverse expanded 1KGP panel (Figure 4F), reflecting its mixed Eastern and Western ancestry, which was underrepresented in the WBBC HRP [114]. Overall, target populations that closely match those in the HRPs demonstrate superior imputation accuracy, particularly when the target population is homogeneous, or its genetic diversity is well-represented in large multi-ancestry reference panels.

## Benefits and applications of genotype imputation

### Genomic medicine and statistical genetics

Complex disorders related to Mendelian diseases and non-communicable diseases substantially contribute to the healthcare burden, primarily driven by a combination of polygenic genetic architecture and diverse environmental risk factors. GWASs have elucidated the complex relationships between genotype and phenotype [115], identifying numerous SNPs linked to common complex diseases and traits; however, these variants explain only a fraction of the observed genetic variance or heritability. Rare SNP mutations or SVs may contribute to the missing heritability. Early applications of phasing and imputation innovations focused on GWAS-based medical discoveries, aiming to dissect heritability by increasing SNP density, enhancing genomic coverage in meta-analyses, and fine-mapping causal variants [11,36,38]. Low-coverage WGS and microarray-based genotyping have proven to be cost-effective and reliable approaches when combined with genotype imputation based on population-specific HRPs.

Recent large-scale GWASs or multi-ancestry GWAS meta-analyses via genotype imputation have demonstrated that combining new genomic resources with imputation strategies significantly expands the pool of available genetic variants, enhancing association signals, facilitating the identification of causal variants, and enabling the meta-analysis of multiple cohorts [5,11,14,39,40,97,110]. Rigorous quality control practices for SNP data in GWASs have been established to ensure data accuracy and reliability [116,117]. The UK Biobank (UKB) cohort, encompassing approximately 500,000 individuals, represents a crucial resource for well-powered GWASs on various quantitative traits [118], including body mass index [119], type 2 diabetes mellitus [120], and major depressive disorder [121]. Notably, Gaynor et al. highlighted that single-variant and gene-based association analyses using WES combined with imputed array data yield signal detection rates within 1% of those achieved with WGS data [122]. Yang et al. conducted single-variant testing and variant-set analysis on East Asian-based GWAS for hip and femoral neck bone mineral density traits, identifying the SEAD-imputed rare variant rs60103302 near *SNTG1* as associated with hip bone mineral density. Similarly, the GEL-imputed UKB GWAS revealed numerous rare trait-associated variants [39]. These newly identified causal variants offer valuable insights into the genetic architecture of human diseases or traits, allowing for the stratification of individuals at elevated risk for specific diseases. Polygenic risk score reconstruction can be performed to estimate genetic predispositions, incorporating individual variability in relevant quantitative traits [123]. This knowledge has contributed to improved patient outcomes through early detection, prevention, and targeted treatment strategies.

### Population genetics

The reconstruction of genetic architecture in ethnolinguistically diverse groups in population genetics has proven crucial for understanding human origins, evolution, migration, and admixture history [124]. However, limited access to resources and financial constraints have historically restricted sampling and genotyping efforts [125]. The application of HRPs and imputation techniques has mitigated these limitations, facilitating the collection of more extensive genomic diversity data. Population-specific HRP-based genotype imputation has enabled the accurate reconstruction of missing genotype data, enhancing dataset completeness and providing higher-resolution data for downstream analyses of admixture modeling, biological adaptation, archaic introgression, and medical relevance interpretation [126]. Moreover, imputation increases marker density in integrative genomic datasets, thereby enabling more statistically robust analyses of mutation, recombination, natural selection, genetic drift, and gene flow. This process transforms individual genotype data into shared haplotypes derived from multiple reference populations, reducing or correcting false positives or negatives caused by population stratification [127]. As a result, fine-scale reconstructions of population evolutionary processes are possible, elucidating demographic histories among diverse populations and establishing a foundational framework for precision medicine systems [128]. Borda et al. evaluated haplotype discrepancies between genotyping-only and imputed datasets by calculating IBD segments. Their findings demonstrated that using exclusively imputed data for IBD analysis did not introduce bias for segments exceeding four centimorgans. Leveraging imputed data, this work provided a detailed depiction of fine-scale population structure, recent gene flow, and long-distance migration across Latin America [129]. Overall, the availability of high-quality genomic resources has dramatically advanced the study of human evolutionary history, facilitating the use of sophisticated algorithms and tools to analyze high-density DNA sequence variation and offering deeper insights into complex evolutionary processes [130].

### Pharmacogenomics

Precision medicine aims to understand individualized disease progression and treatment responses. Genetic variations may influence susceptibility to specific diseases, either increasing or reducing risk, and they also affect responses to certain medications, a field known as pharmacogenomics [131]. Pharmacogenomics explores how genetic variation impacts individual drug responses, particularly in relation to absorption, distribution, metabolism, and excretion (ADME) processes. Given the increasing costs and slow pace of new drug discovery, there has been growing interest in drug repurposing, the practice of adapting existing drugs to treat both common and rare diseases [132]. Studies have indicated that genes identified through GWAS as being associated with disease traits are more likely to encode druggable proteins than other genomic regions [133]. Genotype imputation, utilizing population-specific HRPs, has been applied to estimate missing genomic data and predict individual drug responses, such as the *HLA*-B*15:02 variant related to carbamazepine responses and other genes related to clopidogrel, peginterferon, and warfarin reactions [74]. This approach facilitates the development and optimization of personalized drug therapies, identifying potential drug targets and advancing the understanding of drug mechanisms [134].

### Prenatal screening

The integration of ultra-low-depth sequencing data from non-invasive prenatal testing (NIPT) with genotype imputation leveraging population-specific HRPs has increased the coverage, resolution, and overall comprehensiveness of genotype data for prenatal screening [60,135]. Improvements in genotype imputation performance have been observed with increasing NIPT sequencing depth and the expansion of reference panels [107]. Additionally, WGS on a fetus has been applied to identify potential conditions that may manifest during infancy or childhood, with the objectives of prevention, treatment, or preparation for the child’s arrival [136]. Imputation has proven valuable in genetic prediction and counseling, enriching the understanding of familial genetic histories, predicting disease risk, and facilitating personalized recommendations. Moreover, more precise association studies of complex diseases have been conducted for prenatal genetic diagnosis. Sequencing of fetal DNA allows parents to assess potential health risks, thereby supporting informed decisions regarding pregnancy continuation or termination [137]. The potential transition of genomic sequencing from a specialized test to a broadly accessible healthcare resource will necessitate the collection of high-quality outcome data from large cohorts and sustained efforts in monitoring screening program effectiveness [5]. Achieving these aims in an evidence-based, equitable, and sustainable manner remains essential for safeguarding the well-being of newborns.

### Paleogenomics

The analysis of aDNA extracted from fossils and ancient hominin remains has significantly reshaped human genetic history. However, accurate genotype determination from ancient genomes remains challenging due to limited sequencing depth caused by DNA degradation and microbial contamination [138,139]. Consequently, pseudo-diploids are often employed in population genetic and medical studies, leveraging genomic variations rather than true diploids. Despite these difficulties, evidence has shown that imputation using HRPs constructed from modern humans with similar ancestry represents a reliable method for enhancing aDNA studies within the diploid-based research paradigm, even for populations with coverage depths as low as 0.5× [8]. Martiniano et al. first used imputation and haplotype-based methods in aDNA research, imputing genome-wide diploid genotypes from 14 Middle Neolithic to Middle Bronze Age individuals from Portugal, which identified close relationships between local hunter-gatherers and later Iberian Neolithic populations [140]. Eske et al. analyzed over 5000 imputed ancient genomes from western Eurasia, uncovering genetic changes driven by admixture among ancient steppe pastoralists, agriculturalists, and hunter-gatherers [141]. Their findings highlighted a heightened genetic predisposition to multiple sclerosis, introduced by steppe pastoralists [142], possibly as a selective adaptation to livestock-borne pathogens. The height disparities observed between northern and southern Europeans were linked to varying levels of steppe ancestry. Neolithic farmer ancestry was enriched with risk alleles for emotional traits, whereas Western hunter-gatherer lineages presented a higher prevalence of alleles linked to diabetes and Alzheimer’s disease. Additionally, alleles for lactase persistence emerged in Europe approximately 6000 years ago [143]. Ringbauer et al. developed the ancIBD tool, further advancing the use of imputed aDNA in ancient human history reconstruction [144]. The complete diploid ancient genome offers critical insights into the origins and spatiotemporal evolution of human diseases and traits, elucidating the influence of migration, admixture, and natural selection on disease emergence and development. These findings provide new perspectives on disease evolution and potential therapeutic strategies. With the exponential increase in sequenced ancient genomes, a greater representation of diverse ancestries and time periods is anticipated, offering a unique opportunity to expand HRPs via ancient genomes and standardize imputation methodologies.

### Forensic science and forensic investigative genetic genealogy

Degraded forensic samples often hinder the generation of high-quality, high-coverage genomes required for forensic applications [145]. Genotype imputation utilizing population-specific HRPs has been widely applied in forensic genetic genealogy. In specific criminal cases, degraded DNA biomaterials may limit data availability. The imputation of missing loci enhances the comprehensiveness of genotype profiles, improving parentage testing, individual identification, forensic phenotype prediction, biogeographic ancestry inference, and phylogenetic reconstruction. This method enhances the accuracy of DNA comparisons, aiding in the identification and familial relationship determination of criminal suspects [146]. A notable example underscoring this approach’s impact is the Golden State Killer case. Despite raising privacy concerns and sparking intense debate within the academic community [147,148], forensic genealogy has demonstrated significant potential in resolving cold cases and bringing perpetrators to justice [149]. Its utility extends to identifying missing persons, locating relatives, and identifying human remains.

Population-specific, high-quality HRPs have shown tremendous value across diverse fields, including human evolutionary studies, disease genomics, pharmacogenomics, prenatal screening, paleogenomics, and forensic science. Continual updates to reference panels and imputation algorithms remain critical to maintaining the precision of imputed genotypes and advancing both research and clinical applications.

## Challenges and perspectives

### Inclusion of underrepresented ethnolinguistically diverse populations in the HRPs

**Figure 5:**
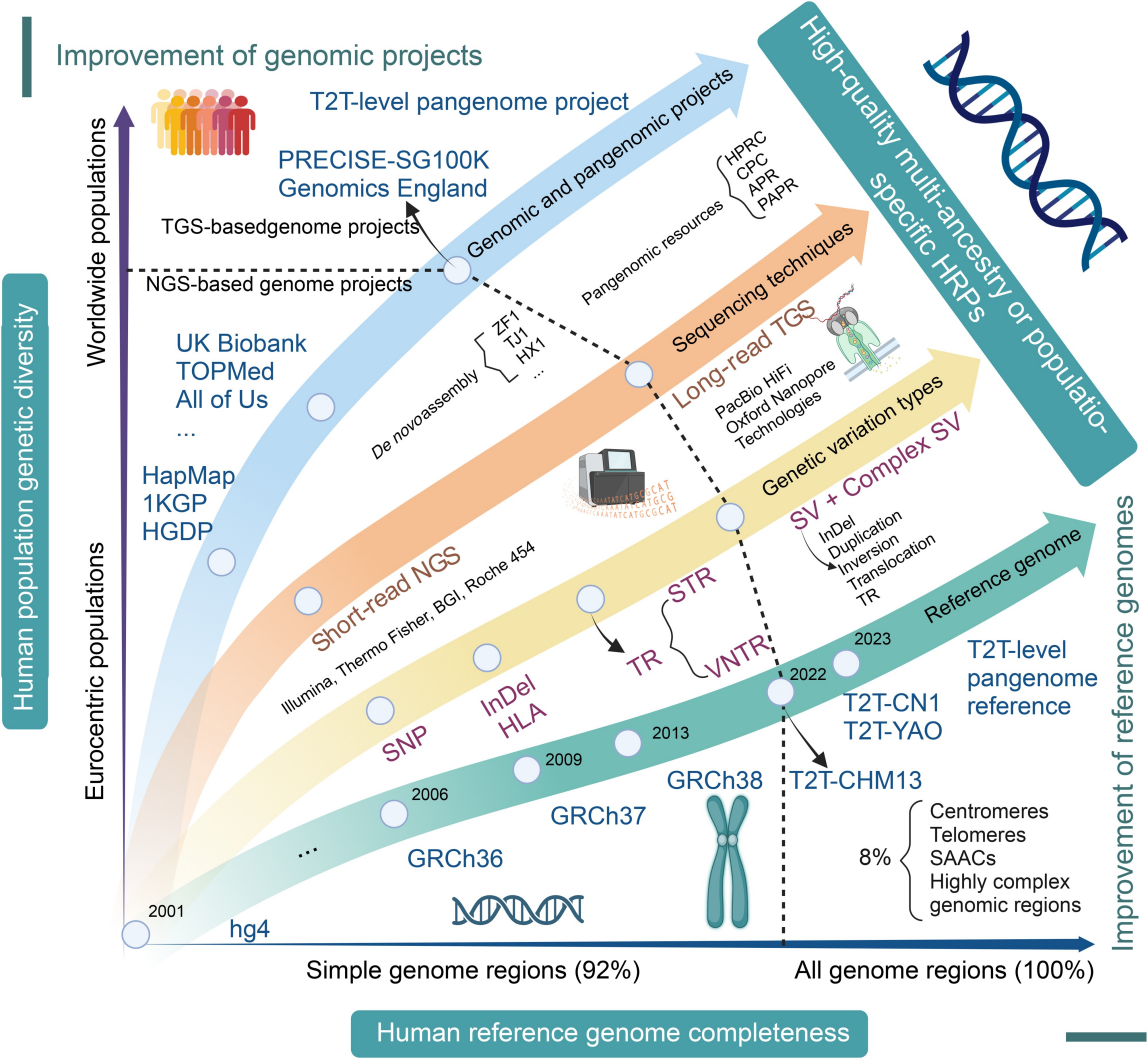
Development and enhancement of the human reference genome, genome projects, and HRPs in the genomic and pangenomic eras We summarized the “2-3-4-5” rule to present all the past, present, and future of the HRPs. “2” denotes the two dimensions: human reference genome completeness and human population genetic diversity. The X-axis represents the improvement in the completeness of the human reference genome, from 92% to 100%, while the Y-axis represents the enhancement of human population diversity in genomic research, transitioning from being centered on European populations to including all populations. “3” highlights three key goals of the reference genome, human genomic projects, and HRPs: the enhancement of human reference panels, including the creation of a perfect T2T-level pangenome reference; the improvement of genomic projects; and the establishment of high-quality multi-ancestry or population-specific HRPs. The four (“4”) arrows in different colors represent the development of genomic and pangenomic projects, the advancement of sequencing techniques, the increase in the types of genetic variations discovered and analyzed, and the updates to human reference genome versions. The five (“5”) major blocks represent the transition from genomic projects based on NGS technologies to those based on TGS technologies; the shift from single-sample *de novo* assembly to the creation of multi-sample human pangenomic resources; the evolution from short-read NGS platforms to long-read TGS platforms; the expansion from simple SNPs to complex SVs; and the progression from reference genomes with gaps to complete, gap-free reference genomes. Ultimately, by integrating these advancements, we can construct a multi-ancestral or population-specific high-quality HRP. T2T, telomere-to-telomere; ZF1, *de novo* assembly of a Tibetan genome; TJ1, *de novo* assembly of a Tujia genome; HX1, *de novo* assembly of a Han Chinese genome; HPRC, Human Pangenome Reference Consortium; CPC, Chinese Pangenome Consortium; APR, Arab Pangenome Reference; PAPR, Pacific Ancestry Pangenome Reference; NGS, next-generation sequencing; TGS, third-generation sequencing; InDel, insertion and deletion; HLA, human leukocyte antigen; TR, tandem repeat; STR, short tandem repeat; VNTR, variable number of tandem repeats; SV, structural variation; SAAC, short arm of acrocentric chromosome.

The application of high-quality HRPs presents significant opportunities and challenges in the evolutionary genomics and pangenomics eras (**Figure 5**). As precision medicine, population genetics, and evolutionary biology advance, HRPs have become critical tools for improving genotype imputation, ancestry inference, and disease association studies. However, a key challenge lies in the underrepresentation of ethnolinguistically diverse populations in existing human genomic cohorts and the summarized panels. Large-scale, medical-driven human genomics data have primarily been collected from participants in metropolitan areas, often failing to capture the diversity of anthropologically informed local populations [25,39]. Consequently, current panels exhibit biases toward data from a limited number of ancestries, reducing imputation accuracy in underrepresented groups [25]. This shortfall hinders the comprehensive characterization of global genetic diversity, exacerbating health disparities and undermining equitable and personalized healthcare initiatives.

Historically, genomic studies have focused predominantly on populations of European or North American ancestry, perpetuating so-called European bias [150,151]. Our findings demonstrate that 60.7% of high-quality HRPs derived from European descendants inadequately represent non-European genetic variants, resulting in imprecise imputation. To address this issue, regional genome projects have been launched to construct population-specific HRPs, increasing diversity to some extent. However, the lack of internationally standardized sequencing and data-processing guidelines has introduced inconsistencies, complicating joint analyses and masking true signals. The Global Alliance for Genomics and Health has sought to address these challenges by promoting collaboration and interoperability, aiming to develop a standardized framework for capturing human genetic diversity.

### High-quality genomic infrastructure and bioinformatics facilitating data sharing

Generating high-quality, population-specific HRPs requires large-scale, high-resolution sequencing data, which remains costly and resource-intensive. The need for robust data curation, quality control, and significant computational resources further complicates the development and maintenance of these panels. Key foundational elements include biorepositories and computing infrastructure, funding strategies, capacity building, global consortia cooperation, and stakeholder will from research and funders. Data sharing and equity have been longstanding points of debate in genomic research and reference panel merging and optimization, as the availability of genomic data plays a pivotal role in advancing precision medicine and personalized treatments [152]. Currently, the common formats for sharing raw genomic data can be categorized into two types: intra-federation sharing and cloud-based sharing. For example, data from the UKB have attracted global scientific interest, with its sharing through cloud-based platforms, significantly advancing genomic research in the UK. Similarly, initiatives such as TOPMed and All of Us have expanded data sharing among their members. The International Hundred Thousand Plus Cohort Consortium has brought together over 100 cohorts from 43 countries, encompassing more than 50 million participants [153].

However, unrestricted data sharing is not universally appropriate. In 2016, representatives from academia, industry, funding agencies, and scholarly publishers established the FAIR Data Principles, emphasizing data that are findable, accessible, interoperable, and reusable [154]. In 2019, the Global Indigenous Data Alliance introduced the CARE Principles, focusing on collective benefit, authority to control, responsibility, and ethics in indigenous data governance [155]. Genomic data sharing must adhere to both FAIR and CARE principles to maximize value while addressing ethical, legal, and social implications. Advanced algorithms are essential to enable secure sharing, evaluation, and selection of genomic data. For example, the Recombine and Share Haplotypes method generates synthetic HRPs by simulating hypothetical descendants of reference panel samples after a user-defined number of meiosis events [156]. Meta-imputation offers another approach, leveraging multiple imputation servers based on different reference panels to improve the accuracy of target sample imputation [114]. These strategies partially integrate the benefits of multiple HRPs while addressing privacy concerns.

Although numerous HRPs have been generated, only a limited number are publicly accessible (Figure 2). Enhanced data accessibility could facilitate reciprocal imputation approaches, integrating the strengths of multiple HRPs. Achieving a balance between data utility and privacy preservation requires collaboration among the technical, regulatory, and ethical communities. Experts in computer security, genetics, computer science, ethics, and privacy law must work together to establish policies that support efficient, privacy-respecting genomic data sharing [157].

### T2T-level HRPs and multi-variant integration

The advent of large-scale genomic datasets [158,159], the completion of the T2T human genome sequence [160,161], and the development of human pangenome projects [162,163] have underscored the importance of comprehensively capturing human genetic diversity and elucidating the functional roles of population-specific sequences and variations (Figure 5). The T2T reference genome and pangenome-based third-generation sequencing paradigm have provided geneticists unprecedented opportunities to develop high-quality HRPs with multiple ancestries and genetic variant types. In the T2T and pangenomic eras, integrating complex SVs and rare haplotypes into HRPs introduces additional complexity, requiring sophisticated algorithms and analytical approaches. Technological advancements, particularly in long-read sequencing and phased genome assembly, have enabled significant progress in refining HRPs. Improved methodologies for resolving complex haplotypes and rare alleles are essential for enhancing panel specificity and accuracy. Collaborative efforts across global consortia and ongoing algorithmic innovations will be key to addressing existing limitations.

Empirical studies on the impact of rare variants on complex traits and diseases remain limited, largely due to challenges in genotype imputation for rare variants, compounded by European bias, small sample sizes, and inadequate sequencing depth [38]. Previous HRPs predominantly included SNPs but rarely incorporated InDels or SVs, largely due to challenges in high-quality SV calling and phasing. This absence has severely constrained downstream analyses, as complex variations undetected by SNPs likely account for a significant portion of unexplained heritability [164]. To address these limitations, the expanded 1KGP has improved HRPs by integrating high-confidence InDels and SVs [42]. However, short-read WGS methods are limited in detecting SVs in highly repetitive genomic regions compared with long-read sequencing approaches. Recent efforts, such as SNP/short tandem repeat (STR) combined panels, have enabled genome-wide STR imputation [165,166]. However, current imputation tools remain optimized solely for SNPs, highlighting the need for further improvements to accommodate other variant types.

The future objective for HRPs is to establish a pangenome HRP based on the T2T assembly. Such advancements will enable large-scale cohort studies in precision medicine, encompassing targeted prevention, treatment, and diagnosis. High-quality HRPs hold transformative potential across diverse fields, including paleogenomics, forensic science, pharmacogenomics, and clinical diagnostics. Expanding and diversifying these panels while maintaining stringent quality standards is essential to maximize their impact. Addressing these challenges will foster more equitable research outcomes, drive biomedical innovation, and deepen our understanding of human genetic diversity. Despite these challenges, haplotype-resolved reference panels offer significant opportunities to advance precision medicine, enhance the detection of disease-associated variants, and provide deeper insights into population histories. Future efforts should prioritize emerging technologies, such as long-read sequencing and artificial intelligence-driven bioinformatics tools, to overcome existing limitations. Collaboration across scientific, medical, and indigenous communities will be crucial to ensuring the quality, inclusivity, and ethical application of HRPs in the genomic and pangenomic landscapes.

### The trade-off between multi-ancestry integrative HRPs and population-specific HRPs

Multi-ancestry integrative HRPs incorporate high-quality genomic data from individuals of diverse genetic backgrounds, often spanning multiple continents or genetically distinct subgroups within a continent, as exemplified by the state-of-the-art TOPMed reference panel [1]. TOPMed-based imputed datasets identify more variant sites and high-impact consequence variants than those generated from other panel-imputed datasets, highlighting the potential of multi-ancestral reference panels to provide more comprehensive genotypic information in the context of mixed and heterogeneous target populations with different ancestral sources. In contrast, population-specific panels focus on genomic data from relatively homogeneous populations with well-characterized genetic backgrounds, such as the NyuWa, ChinaMAP, and WBBC panels, which are optimized for imputation within Han Chinese populations [4,21,63].

Several key factors, including haplotype diversity, panel size, genotype imputation accuracy, preservation of the LD structure, privacy and data sharing considerations, adaptability, and hardware requirements for big data processing, influence the choice between these panel types. Multi-ancestry panels capture a broader spectrum of genetic variation, making them crucial for studies involving globally diverse populations. By integrating data from multiple populations, these panels offer a more comprehensive view of haplotype diversity. However, population-specific panels, which focus on a single group, typically achieve higher resolution and more precise genetic insights for that population. They present greater genotype imputation accuracy, especially for rare variants, as they are optimized to reflect unique genetic variants and LD patterns. In contrast, multi-ancestry panels, while accommodating a range of heterogeneous populations, may suffer from reduced accuracy for specific groups due to the need to balance genetic variation across heterogeneous datasets. These panels also face challenges in preserving LD structure, as LD patterns differ significantly between populations. Population-specific panels, by concentrating on a single group, better maintain the characteristic LD structure, thereby increasing the resolution of population-specific analyses.

From a privacy perspective, multi-ancestry panels offer enhanced protection by blending haplotypes across diverse populations, reducing the risk of directly linking genotypes to phenotypes and facilitating broader data sharing while safeguarding individual privacy. In contrast, population-specific panels may require additional privacy measures due to their targeted design. Despite this, population-specific panels provide fine-grained insights into genetic studies within a specific population, making them ideal for tailored research. Multi-ancestry panels, on the other hand, are better suited for cross-population analyses and global studies, offering broader applicability. Both multi-ancestry and population-specific HRPs present unique advantages and challenges. The selection of an appropriate panel should align with the imputed objectives, the genetic characteristics of the target population, and privacy requirements. A flexible approach, leveraging one or both panel types based on research needs, may maximize the utility of HRPs across diverse genetic studies.

## CRediT author statement

**Qingxin Yang:** Formal analysis, Methodology, Writing – original draft, Visualization, Writing – review & editing. **Yuntao Sun:** Visualization. **Shuhan Duan:** Investigation. **Shengjie Nie:** Project administration. **Chao Liu:** Project administration, Supervision. **Hong Deng:** Funding acquisition, Writing – review & editing. **Mengge Wang:** Funding acquisition, Supervision, Writing – review & editing. **Guanglin He:** Conceptualization, Writing – review & editing, Resources. All authors have read and approved the final manuscript.

## Competing interests

The authors have declared no competing interests.

## Supplementary Material

qzaf022_Supplementary_Data
