# Supplementary material for: High-quality Population-specific Haplotype-resolved Reference Panel in the Genomic and Pangenomic Eras
Source: Genomics Proteomics Bioinformatics. 2025 Mar 10;23(6):qzaf022. doi: 10.1093/gpbjnl/qzaf022 (PMC13175255; doi:10.1093/gpbjnl/qzaf022)
Supplement: qzaf022_Supplementary_Data [file qzaf022_supplementary_data.zip › supplementary material captions.docx]

**Supplementary material**

**Table S1 All characteristics and details of all HRPs**

**Table S2 Independent validation results for phasing and imputation tools**
